# Insights and challenges on animal models for progressive multiple sclerosis

**DOI:** 10.1186/s12974-026-03805-3

**Published:** 2026-04-15

**Authors:** Isabella F. Nalepa, Nicolai O. Haahr, Vasileios Bekiaris, Martin R. Jakobsen, Ayodeji A. Asuni, Anouk Benmamar-Badel

**Affiliations:** 1 Preclinical Fluid Biomarkers and Occupancy, H. Lundbeck A/S, Valby, Denmark; 2 Department of Health Technology, Technical University of Denmark, Kgs Lyngby, Denmark; 3 Department of Biomedicine, Aarhus University, Aarhus, Denmark

**Keywords:** Animal Models, Progressive Multiple Sclerosis, Neuroimmunology, Experimental Autoimmune Encephalomyelitis, EAE, Cuprizone, Lysolecithin, Theiler’s murine encephalomyelitis virus

## Abstract

Progressive multiple sclerosis (PMS) remains a major clinical challenge due to its complex pathophysiology and limited therapeutic options. While disease-modifying therapies have significantly improved prognosis for relapsing forms of multiple sclerosis (RMS), effective treatments for the progressive, neurodegenerative phase are lacking. Animal models have been instrumental in elucidating the molecular mechanisms underlying multiple sclerosis (MS) pathology and have guided the drug development field for MS, yet their ability to faithfully replicate the development of PMS remains debated. The most frequently used animal models are either immune-mediated, viral, or toxin-induced demyelinating models, but none of these are able to fully mimic the complexity of PMS pathology. This review examines the immunopathological features of animal models of PMS and critically evaluates their strengths and limitations for studying the progressive stages of disease. We highlight the variability that currently exists in the experimental autoimmune encephalomyelitis (EAE) field, as methodologies vary between EAE model paradigms which limit comparability. We conclude that no single model recapitulates PMS, however, refined and combined approaches, with careful attention to strain, age, sex, and induction protocol, can improve translational relevance and support development of therapies targeting PMS.

## Background

Multiple Sclerosis (MS) is an autoimmune disease of the central nervous system (CNS), whereby chronically activated immune cells lead to progressive demyelination. It affects over 2.9 million people worldwide, with a prevalence in women more than twice as high as in men [1].

The disease onset is typically observed in young adults between 18 and 40 years of age. The majority (~ 85%) of patients initially exhibit a relapsing–remitting phenotype (RRMS), characterized by alternating episodes of recurrent neurological symptoms (relapses) and subsequent partial or complete recovery (remissions). The first relapse or indication of neurological deficit is referred to as clinically isolated syndrome (CIS). Twenty years after onset, the majority of these patients will have transitioned to a secondary stage of the disease, marked by few or no remissions and progressive accumulation of disabilities (secondary progressive MS; SPMS). RRMS has a female preponderance with a female to male ratio of approximately 3:1 [2, 3]. However, some studies suggests that the men that do get diagnosed with RRMS present with a faster disease progression compared to women [4, 5]. The remaining 15% of patients present with primary progressive MS (PPMS) from the onset of the disease, which is characterized by immediate and gradual symptom progression and lack of classical relapses and remissions [6–8]. Interestingly, the sex-bias diminishes for PPMS where the sex-ratio is almost equal [4, 9]. Table 1 provides a comprehensive overview of the nomenclature associated with MS.

**Table 1:** MS nomenclature: different categorizations of MS

The clinical course of MS is typically divided into four categories, standardized and agreed upon by international experts in 2013[10]:
*Clinically isolated syndrome (CIS)*: The initial clinical manifestation of a disease exhibiting features of inflammatory demyelination that may suggest MS but does not yet meet the criteria for dissemination in time
*Relapsing–Remitting MS (RRMS)*: Characterized by attacks or episodes of new or worsening symptoms (relapses) followed by periods of partial or complete recovery (remissions)
*Secondary Progressive MS (SPMS)*: Develops in individuals who initially had RRMS and is marked by a gradual worsening of symptoms and disability over time, with or without relapses
*Primary Progressive MS (PPMS)*: Characterized by a steady progression of symptoms from the onset, without distinct relapses or remissions [10]
At times the description of disease course can be integrated into two broader categories:
*Relapsing MS* (RMS) is a category that includes CIS, RRMS, and active SPMS
*Progressive MS (PMS)* includes PPMS and inactive/worsening SPMS and is characterized by a continuous progression of disability [10]
More recently, the focus has shifted to distinguish between how patients acquire MS disability, when categorizing different types of MS
*Relapse-associated worsening (RAW)*: A gradual accumulation of disability resulting from incomplete recovery after a relapse
*Progression Independent of Relapse Activity (PIRA)*: Worsening of disability in MS patients that occurs independently of relapses [11, 12]
While the RAW and PIRA classifications are gaining recognition within the field of MS research, we have chosen to use the nomenclature of RRMS, SPMS, PPMS, RMS, and PMS in this review of animal models for PMS

From a neuropathological perspective, a diagnostic hallmark of MS is the presence of large demyelinating lesions in cortical white matter (WM) and grey matter (GM), in the deep brainstem nuclei, and, to a lesser extent, in the spinal cord [13]. These lesions ultimately result in neurodegeneration and brain atrophy, and clinical manifestations such as motor weakness, loss of balance, sensory deficits including vision loss or double vision, and cognitive decline [14–16]. The etiology of MS is yet to be completely elucidated, but it is believed to result from a combination of environmental factors and genetic predisposition [7]. Although no curative treatment exists, numerous disease-modifying therapies (DMTs) have been approved [17, 18]. These therapies aim to reduce the frequency and severity of relapses by suppressing or modulating immune responses, thereby alleviating the associated clinical symptoms. However, while several DMTs reduce inflammatory activity and can slow disability accumulation in some progressive MS populations, progression and neurodegeneration remain important unmet needs [19–21]. Most DMTs are approved for treatment of relapsing MS (RMS) [17], a smaller number have shown benefit in some SPMS populations [19, 22, 23], but only one DMT has shown efficacy for treatment of PPMS, underlining the unmet need of treatment options for these patients [20] (Table 2).

**Table 2:** FDA-approved MS therapies—mechanisms, indications, and EAE model efficacy

Generic Name	Mechanism of Action	EAE Model Efficacy	Indication	Clinical Human Data	EAE Model Data
Ofatumumab	Anti-CD20 mAb	No data. Data on surrogate anti-CD20 mAb and mouse-human chimera (Rituximab show effects in huCD20 C57Bl/6)	RMS	[97]	[98]
Ocrelizumab	Anti-CD20 mAb	No data. Data on surrogate anti-CD20 mAb and mouse-human chimera (Rituximab show effects in huCD20 C57Bl/6)	RMS, PPMS	[20]	[98]
Ocrelizumab hyaluronidase-ocsq	Anti-CD20 mAb	No data	RMS, PPMS	[99]	-
Natalizumab	α4β1 integrin inhibitor	Yes when using the murine anti-VLA4. Effect in C57Bl/6:MOG_35–55_ and in guniea pig EAE	RMS	[100]	[101, 102]
Ublituximab	Anti-CD20 mAb	No data	RMS	[103]	-
Alemtuzumab	Anti-CD52 mAb	Yes when using the murine anti-CD52. Model dependent	RMS	[104]	[105–107]
Mitoxantrone	DNA intercalator	Yes in rat EAE	RMS	[108]	[109, 110]
Monomethyl fumarate	Nuclear factor (erythroid-derived 2)-like 2 pathway inhibitor	Yes in RR-EAE in SJL mice	RMS	[111]	[112]
Dimethyl fumarate	Nuclear factor (erythroid-derived 2)-like 2 pathway inhibitor	Yes in C57Bl/6 mice	RMS	[113]	[114, 115]
Diroximel fumarate	Nuclear factor (erythroid-derived 2)-like 2 pathway inhibitor	No data	RMS	[116]	-
Interferon β−1a	Not completely elucidated	Moderate effects. Model dependent	RMS	[117, 118]	[119–122]
Interferon β−1b	Not completely elucidated	Yes. Direct effect only shown in rats. Effect of interferon-β is only evident in B-cell independent EAEs	RMS, active SPMS	[123]	[120, 124]
Peginterferon β−1a	Not completely elucidated	No data	RMS	[125]	-
Cladribine	Not completely elucidated	Yes in C57Bl/6:MOG_35–55_	RMS	[126]	[127]
Teriflunomide	Pyrimidine synthesis inhibitor	Yes. Model dependent	RMS	[128]	[129, 130]
Siponimod	Sphingosine-1-phosphate receptor modulator	Yes. Model dependent	RMS, active SPMS	[19]	[131–134]
Ponesimod	Sphingosine-1-phosphate receptor modulator	Yes in C57Bl/6:MOG_35–55_ and Lewis:MBP	RMS	[135]	[136]
Fingolimod	Sphingosine-1-phosphate inhibitor	Yes	RMS	[137]	[138, 139]
Ozanimod	Sphingosine-1-phosphate receptor modulator	Yes in C57Bl/6:MOG_35–55_	RMS	[140]	[141]
Glatiramer acetate	Not completely elucidated	Yes in C57Bl/6 and SJL mice	RMS	[142]	[143–145]

FDA-approved disease-modifying therapies for multiple sclerosis (MS), their primary mechanisms of action, licensed indications, and evidence of efficacy in experimental autoimmune encephalomyelitis (EAE) models. “EAE model efficacy” qualitatively summarizes whether beneficial effects have been demonstrated in EAE models, illustrating the broad use and importance of EAE for development of MS therapeutics. “Clinical human data” and “Preclinical EAE model data” provide representative references when available

*Abbreviations: EAE* Experimental autoimmune encephalomyelitis, *mAb* Monoclonal antibody, *MBP* Myelin basic protein, *MOG* Myelin oligodendrocyte glycoprotein, *MS* Multiple sclerosis, *PPMS* Primary progressive multiple sclerosis, *RMS* Relapsing forms of multiple sclerosis including SPMS; RR-EAE, relapsing–remitting EAE; SPMS, secondary progressive multiple sclerosis

Different animal models of MS recapitulate different aspects of the disease and have significantly contributed to our understanding of its pathogenesis and to the preclinical validation of treatments. Most animal models focus on acute inflammatory phases that resemble RRMS, whereas the chronic inflammation, axonal degeneration, and compartmentalized immune responses characteristic of progressive multiple sclerosis (PMS) are insufficiently captured. This limitation can possibly contribute to the lack of translation of experimental findings into effective treatments for progressive disease [24–26]. The most widely used model of MS is the experimental autoimmune encephalomyelitis (EAE) model, where an immune response against myelin is triggered, causing CNS inflammation, demyelination, axonal damage, and physical symptoms of ascending paralysis. EAE is most often induced in rodents, primarily mice, but can also be induced in large animals such as non-human primates (NHP). Theiler’s murine encephalomyelitis virus (TMEV) relies on viral infection to cause chronic demyelination in mice. The cuprizone and lysolecithin models are toxin-based models that induce demyelination followed by remyelination once the toxin is removed. Each model thus simulates certain features of the disease but does not replicate the complete human pathology. It is therefore crucial to be cautious about selecting the model best suited for the specific aspect of disease under investigation [27].

In this review, we highlight the advantages and limitations of different animal models as tools to investigate pathological mechanisms linked to progressive forms of MS (SPMS and PPMS; hereafter collectively referred to as PMS). We use a feature-driven framework, evaluating each model against the specific PMS relevant disease features it is intended to capture and explain. We therefore first outline key immunopathological features of RMS and PMS to establish benchmarks for model selection and interpretation. We then review the major available models, with a particular focus on murine EAE, as it remains the most widely used platform in preclinical MS research. Finally, we compare the extent to which selected models reproduce PMS-like features, highlight limitations of current approaches, and discuss refinements that could improve translational relevance and support the development of therapies targeting the progressive and neurodegenerative aspects of PMS.

## Clinical features of MS

Accurate modeling of autoimmune diseases in animals requires a thorough understanding of the immunopathological mechanisms underlying the human condition and of the constraints imposed by interspecies differences in immune function and pathology. MS is a disease with different stages and the immunological landscape changes as the disease progresses. During RRMS, the pathology is largely driven by a migration of various types of immune cells from the periphery, across a disrupted blood–brain barrier (BBB) and into the CNS, whereas PMS is characterized by compartmentalized inflammation behind a relatively intact BBB [28–30].

Although the immunopathology of (RR)MS has been extensively reviewed elsewhere [13, 31–35], we will summarize its main features. A hallmark of MS pathology is focal demyelinating plaques. In RRMS, these lesions are predominantly active, which is defined by a great presence of infiltrated monocyte-derived macrophages (hereunder microglia) containing degraded myelin products, which further underlines the importance of infiltration to drive this stage of disease [36]. In line with this, the most infiltrating immune cells reported in active RRMS lesions are CD8 + T cells and monocyte-derived macrophages, and to a lesser degree CD4 + T cells, B cells and plasma cells [28, 37–40]. Of CNS resident cells, both activated microglia and astrocytes have been strongly implicated in MS pathology, even though astrocytes are less extensively studied [41–44]. Additionally, evidence suggests myeloid leukocytes like neutrophils and monocytes also play a role in disease pathogenesis [45–49]. Albeit being quantitatively outnumbered by CD8 + T cells in lesions, B cells are present in the meninges and perivascular spaces in the brain and contribute to the pathology of MS [28, 50]. This is illustrated by one of the most consistent immunological abnormalities in MS being intrathecal synthesis of immunoglobulin G (IgG), detected as IgG oligoclonal bands in the cerebrospinal fluid (CSF) [51–53]. However, the strong efficacy of anti-CD20 therapies especially in relapsing disease implies that pathogenic B cell functions extend beyond antibody secretion and underline the importance of B cells for MS pathogenesis [54, 55]. Although CD4 + T cells infiltrate the CNS less extensively than CD8 + T cells and monocyte-derived macrophages, CD4 + T-helper (Th) cells remain key contributors to MS pathology. Through cytokine release, they promote inflammatory amplification and can contribute to early BBB disruption. In particular, Th1 cells (IFN-γ–producing) enhance pro-inflammatory myeloid activity and support CD8 + T cell and B cell activation, while Th17 cells (IL-17–producing) promote BBB breakdown and activate microglia and astrocytes. [56, 57]. Changes in the Th1/Th17 ratio have in the past been believed to be majorly implicated in MS pathology [58]. However, this binary paradigm of the importance of balance in Th1 and Th17 has been challenged more recently; leaving space for a more complex picture of functionally plastic and heterogeneous Th subsets [59].

In PMS, both the clinical features and the underlying immunopathology evolve compared to RRMS. However, much like early disease etiology, the mechanisms driving disease progression remain incompletely understood. Yet one well-recognized feature of this stage is that disease activity becomes largely disconnected from systemic immune responses and is instead sustained by chronic activation of CNS-resident cells, including microglia and astrocytes [13, 28, 59, 60]. This is exemplified by the degree of infiltration being drastically reduced in patients with PMS, as the T and B cell count in active lesions, inactive lesions and meninges decreases in both PPMS and SPMS patients compared to RRMS [28]. Nevertheless, in PMS patients, CD8 + T cells are still present in higher numbers than CD4 + T cells, in the perivascular cuffs, lesions, parenchyma, normal appearing white matter, and CSF [50, 61, 62]. Interestingly, while the frequency of B cells declines in lesions from PMS compared to RRMS patients, the number of plasma cells significantly increases, suggesting that infiltrated B cells mature to plasma cells as the disease progresses [28]. Despite this quantitative decline, the importance of B cells for the progression of specifically PPMS is illustrated by the successful lowering of clinical progression and magnetic resonance imaging (MRI) signal following depletion of CD20-expressing cells [20].

Active lesions are rare in PMS, while smouldering lesions become more prominent [13, 18, 36]. These lesions are defined by an inactive demyelinated core with few or no infiltrating cells, surrounded by a rim of chronically activated microglia [28, 63]. However, inactive plaques are the most frequent lesion in PMS, defined by a distinct plaque border with only a few or no macrophages nor activated microglia [36]. MRI detectable iron accumulation in activated microglia and macrophages at the edge of the lesion is a pathological hallmark of smouldering lesions but can also be found in some inactive lesions [64]. Lastly, completely remyelinated lesions – or shadow plaques – are also present in PMS. Although, unlike the other lesion types described, the frequency of shadow plaques does not change across the different courses of disease [36].

A unique and characterizing feature of SPMS is the formation of ectopic lymphoid follicles (ELFs). These are defined as aggregates of cells localized in the subarachnoid space, mainly inside the cerebral sulci, that display several germinal center-like features, but lack the typical structure of lymphoid follicles with a germinal center and a mantle zone [65, 66]. ELFs can contain a multitude of different immune cells, including B cells, plasma cells, T cells, follicular dendritic cells (FDC), follicular T-helper cells (TFH), each playing distinct roles in continued MS pathology [59, 66–68]. Cross-talk between pro-inflammatory B cells and microglia as well as monocyte-derived macrophages induce pro-inflammatory cytokine expression, which may further propagate the CNS-compartmentalized inflammation associated with MS progression [69]. One of the key pathological features associated with ELFs is their close spatial relationship to subpial cortical GM demyelination and neuronal loss. Several studies have shown that ELF-positive SPMS cases exhibit extensive gradients of cortical demyelination extending inward from the pial surface, suggesting that inflammatory mediators released from ELFs contribute to GM injury [67, 70, 71]. While ELFs are only present in ~ 40% of SPMS cases, diffuse meningeal inflammation plays a role in the pathology of PPMS and the remaining 60% of SPMS [70, 72]. More detailed and extensive reviews of both innate as well as adaptive immune cells in PMS have been published elsewhere [59, 73–75].

Understanding the immunopathological features of MS, from peripheral immune cell infiltration in RRMS to the compartmentalized CNS inflammation in PMS, is essential for developing relevant animal models, which in turn can increase the translational value of preclinical drug development. These models must reflect the distinct immune dynamics and lesion characteristics observed across disease stages to be effective tools for studying pathogenesis and evaluating therapeutic strategies [76].

## EAE as an animal model of MS

The EAE model was developed in 1933 following observations of paralysis in monkeys vaccinated against rabies using emulsions of rabbit brain [77, 78]. Later reports described a correlation between disease incidence and myelin concentration in the rabbit brain extract leading to refinement of induction protocols for the EAE model [79, 80]. EAE is a broad term to describe any model where an autologous immune response against the myelin sheath is used to induce motor symptoms in an animal. The EAE model has been developed in various species but is mainly used in mice, which is likely due to the plethora of well-studied, highly characterized wild-type strains together with the many established transgenic and knock-out substrains [81]. The model can be induced either actively or passively, with each approach differing in its immunological mechanisms and technical implementation.

### Active EAE—induced by immunization with neuroantigens

Active EAE is induced by immunization with neuroantigens emulsified in an adjuvant – the most commonly used adjuvant is Complete Freund’s Adjuvant [82, 83], but Toll-like receptor agonists and glucan particles can also be used [84]. Several neuroantigens can be used in the induction of active EAE depending on the species and strain used. The different antigens encompass homogenates of CNS tissue (primarily spinal cord), myelin proteins and peptides, and even astroglial-specific proteins such as S100-β [79, 85, 86].

EAE can be induced in multiple species including mice, rats, monkeys, rabbits, and guinea pigs [87]. The model is mostly used in rodents – especially mice. Depending on the mouse strain the protocol sometimes calls for subsequent administration of a co-adjuvant which is believed to boost the immune response by BBB permeabilization and stimulation of Th1, Th2, and Th17 responses [88, 89]. After immunization, the mice will gradually show symptoms of ascending paralysis beginning at the tail tip and can progress to total paralysis of the hindlimbs and in some cases even forelimbs. The mice are observed and given a clinical score based on their symptoms. The model and its disease course can vary depending on which reagents are used and their concentration, as well as on the mouse strain, substrain, sex, and age.

### Passive EAE – induced by adoptive transfer of encephalitogenic T Cells

To induce passive EAE, encephalitogenic T cells specific for myelin self-antigens are adoptively transferred. An extensive protocol for passive EAE has been published by Tanaka et al. [90]. Passive EAE is a valuable tool to study the effector phase of CNS autoimmunity, offering advantages over active immunization paradigms. In passive EAE, myelin-reactive CD4⁺ T cells are isolated from donor mice, polarized in vitro, and transferred into naïve recipient mice. This approach bypasses the peripheral priming phase, hereby allowing for direct assessment of how effector T cell populations mediate disease [91, 92].

The choice of recipient strain impacts the disease course, severity, and progression, enabling modelling of PMS–like features[92, 93]. Passive EAE has demonstrated that Th17 and Th9 subsets, in addition to classical Th1 cells, can induce CNS autoimmunity, and that the phenotype of transferred cells strongly affects lesion distribution, clinical severity, and pathological patterns [92]. By controlling the composition, activation state, and timing of transferred T cells, this model allows investigation of chronic or cumulative tissue damage analogous to secondary PMS. In this context, passive EAE models have been shown to develop ELF-like structures in the meninges [94, 95].

### EAE as a valuable tool in preclinical MS research

Regardless of whether EAE is induced actively or passively, and despite conflicting opinions on which disease characteristics and pathologies it truly recapitulates, EAE has undeniably been central to elucidating key immunological mechanisms in MS and other demyelinating diseases of the CNS. Consequently, murine models of EAE have been central in preclinical validation of multiple FDA-approved MS therapies [96]. This is exemplified by the fact that most approved immunomodulatory drugs for MS, including glatiramer acetate, fingolimod, and natalizumab, were first validated in EAE before entering clinical trials [79]. These models allow controlled evaluation of treatment effects on neuroinflammation, demyelination, and immune cell infiltration.

Importantly, different EAE variants respond differently to therapeutic classes: for example, myelin oligodendrocyte glycoprotein (MOG)_35–55_-induced EAE mainly reflects T cell–mediated pathology and in general responds well to T cell–modulating agents but has less use for screening B cell-targeted therapies. B cell dependent EAE models such as MOG_1–128_-induced EAE therefore become a valuable tool when investigating B cell targeted therapies [86]. Table 2 summarizes FDA-approved MS treatments, their mechanisms of action, approved MS indications, and evidence of efficacy in EAE models. While many EAE protocols are best suited for modelling RMS, certain variants and induction protocols have shown relevance to PMS research, supporting its continued use in screening candidate drugs for both disease stages.

**Fig. 1:**
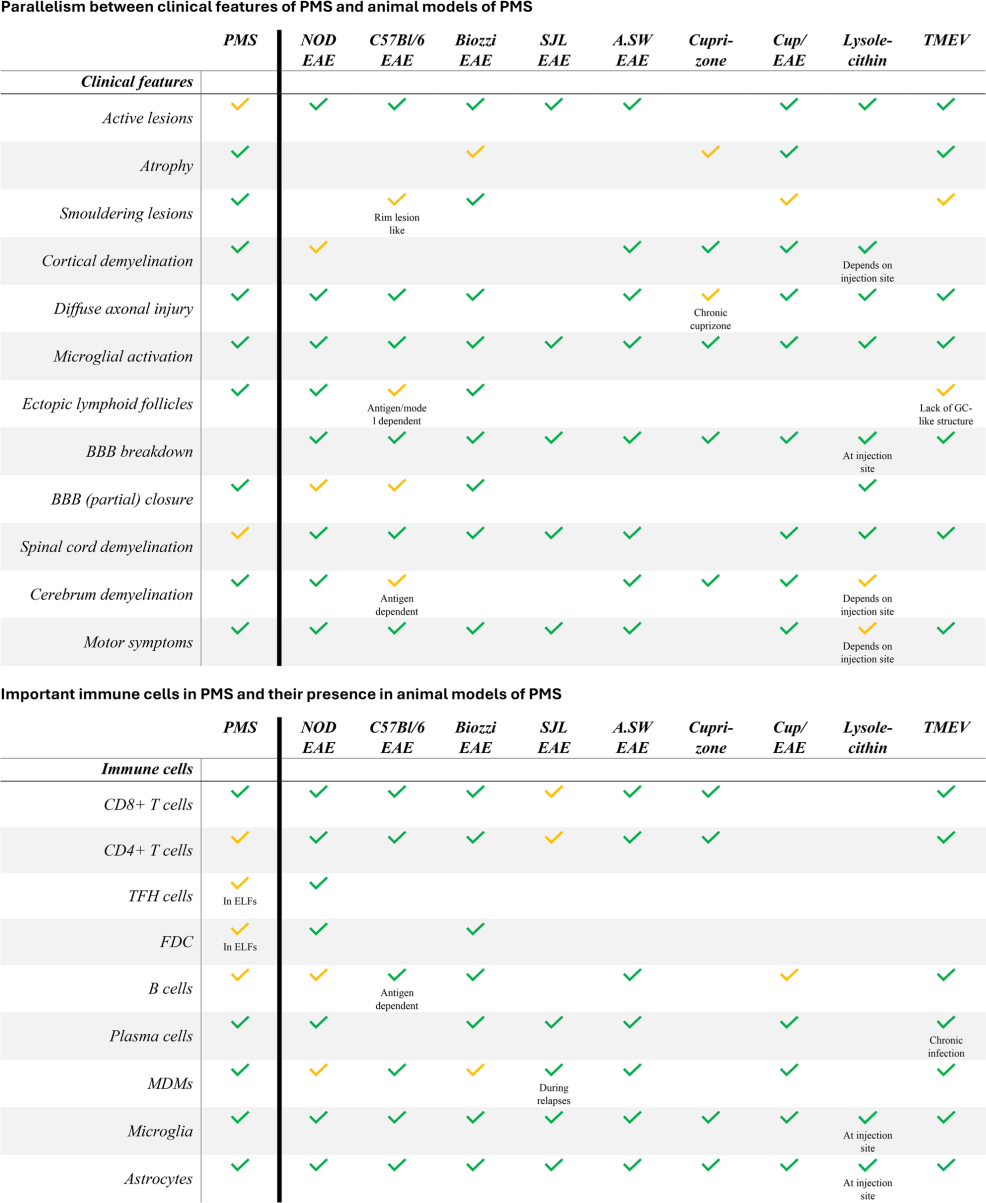
Similarities of clinical features and involved immune cells between PMS and animal models of PMS. Comparing clinical features and prominently involved immune cells in PMS to animal models of PMS. Clinical features and immune cell population in PMS and animal models are indicated as present (green checkmark), potentially present or present in low numbers (yellow checkmark), or not present (empty cell). *PMS, progressive multiple sclerosis; EAE, Experimental autoimmune encephalomyelitis; Cup, Cuprizone, TMEV, Theiler’s Murine Encephalomyelitis Virus; FDC, Follicular dendritic cell; MDMs, Monocyte-derived macrophages*

In this review, we describe an animal model as “progressive” when neurological disability shows sustained worsening over time, either as progression from onset (primary progression) or as a transition from an initial relapsing–remitting phase to persistent disability accumulation (secondary progression); models that stabilize at a chronic severe disability plateau without meaningful recovery are also discussed as progressive-like. Figure 1 summarizes and compares clinical features and the immunological landscape of PMS with the models reviewed.

## PMS-relevant EAE models

### C57Bl/6 mice – acute encephalomyelitis with neuroantigen concentration-dependent disease progression

The C57Bl/6 mouse strain is often used in EAE studies due to its highly inbred status and readily available repertoire of transgenic variants. This strain is however resistant to induction of EAE using proteolipid protein (PLP) and myelin basic protein (MBP) peptides but is susceptible to EAE induced with the MOG_35–55_ peptide or full-length MOG protein [146]. EAE can be induced in both male and female C57Bl/6 mice and is mainly stated to cause an acute monophasic or chronic disease course [147]. Due to this monophasic disease course, C57Bl/6 EAE is said to mimic the initial, acute phase of an MS attack. However, we and others have often observed a slight remission and occasional relapses in C57Bl/6 EAE, which calls for further exploration within the EAE community [89]. The C57Bl/6 EAE model has been found to vary in its disease progression and severity based on the amount of peptide used –– ranging from 50 to 300 µg/mouse, with increasing peptide concentrations shifting the disease towards a primary progressive course [148, 149].

EAE in C57Bl/6 mice shares several pathological features with MS, including WM demyelination, axonal loss, and CNS infiltration of immune cells – especially CD4 + and CD8 + T cells, macrophages, and microglia. The cervical and lumbar part of the spinal cord is affected by demyelination and axonal loss, accompanied by prominent infiltration and lesion formation under the pia mater and throughout the parenchyma [148–151]. EAE in C57Bl/6 mice leads to accumulation of inflammatory T cells around blood vessels in the subarachnoid space and just below pia mater, just as it is found in ELFs in SPMS. Additionally, these mice present with increased microglia/macrophage reactivity around the inflammatory foci when assessed as the number of F4/80 positive cells, which has the potential of modelling the localization of microglia seen in iron-rim lesions found in PMS [149]. Interestingly, it has been shown that male and female mice present with similar disease courses, but that females often have increased infiltration of immune cells to the spinal cord accompanied by increased demyelination compared to males [152, 153].

Under certain conditions (e.g., higher antigen doses, ageing, or using alternative antigens for immunization), the C57Bl/6 EAE model can exhibit chronic or progressive disease courses, with features such as ELFs and B cell involvement that align more closely with PMS [148, 154]. Berard et al. found that higher concentrations of MOG_35–55_, *Mycobacterium tuberculosis*, and pertussis toxin (PTX) shifted the disease course from relapse-remitting EAE (RR-EAE) to chronic EAE (CH-EAE), resulting in a greater lesion burden in the lumbar spinal cord at the late remission/progressive stage of disease [148]. Although this chronic course was accompanied by lower numbers of infiltrating cells to the CNS, there was a noticeable shift in the CD8 + :CD4 + T cell ratio in the CNS, towards an increase in CD8 + T cells at the peak and progressive stages of CH-EAE compared with RR-EAE [148]. This could to some degree resemble the increased CD8 + to CD4 + T cell ratio seen in PMS compared to RRMS. However, it does not reflect the fact that CD8 + T cells outnumber CD4 + T cells in MS lesions in general.

Pathology in the EAE model in C57Bl/6 mice has for a long time been believed to be solely T cell-driven, and independent of B cells, as EAE is inducible in B cell deficient mice [155]. However, IL-10 secreting regulatory B cells have later been shown to affect disease onset [156]. Interestingly, IL-10 levels have been shown to be inversely correlated with disability in PMS patients and been proposed as a promising fluid biomarker for disease burden [157, 158].

If C57Bl/6 mice are immunized with an MBP-PLP fusion protein known as MB4, the outcome is a B –cell-dependent pathophysiology with CD8 + T cell involvement as well as B cell aggregates or ELFs, all hallmarks of PMS pathology [159–161]. Some have found that MB4 induces a progressive disease in which infiltration of the cortex, spinal cord, and cerebellum is dynamic and demyelination is chronic, and the number and size of lesions positively correlate with disease severity [162].

The transgenic C57Bl/6 2D2 MOG_35–55_-specific T cell receptor mice are known to develop EAE spontaneously. After crossbreeding with MOG-specific immunoglobulin (Ig) heavy chain mice, Betelli et al. reported the presence of ectopic lymphoid follicles in the spinal cord and optic nerve [163]. Additionally, in a passive EAE model where MOG-specific Th17 cells from 2D2 mice are transferred to C57Bl/6 mice, ELFs form in the meninges [94, 164]. It has been shown that T and B cell interactions in meningeal ELFs cause reactivation of autoreactive T cells, potentially making this a useful model for studying smouldering inflammation, which is a hallmark of PMS [94].

However, the C57Bl/6 EAE models present notable limitations. Their default disease course is typically monophasic or acute, lacking the slow, continuous progression characteristic of PMS. Astrogliosis is generally absent, and cortical GM involvement is limited. Furthermore, the difference between C57Bl/6 J and C57Bl/6N substrains are often not considered nor indicated in publications, although reports suggest that C57Bl/6 J mice present with more severe EAE compared to C57Bl/6N mice [165]. Moreover, the disease phenotype is highly sensitive to experimental variables such as peptide dose and adjuvant use, which can complicate interpretation and reproducibility. The gut microbiome has also been found to affect disease onset and severity, further complicating reproducibility – especially across different facilities [166]. While transgenic variants can enhance the EAE models relevance to PMS, these modifications introduce artificial elements that are not present in human disease.

### Non-obese diabetic mice – chronic encephalomyelitis with age-dependent onset

EAE in non-obese diabetic (NOD) mice is used to model PMS, as these mice develop chronic encephalomyelitis after MOG_35–55_ immunization [167]. The NOD-EAE model captures several key features of PMS, including a disease course progressing from relapsing–remitting to progressive, earlier onset in older mice, and progressive accumulation of neurological deficits [95, 168, 169]. Pathologically, it mirrors human PMS through widespread demyelination and axonal loss, particularly in the brain [168]. MRI analyses have shown increased demyelination and axonal loss in the fimbria, internal capsule, and corpus callosum and have also revealed BBB disruption in the fimbria, internal capsule, periventricular zone, and cerebral peduncle [168]. Additionally, immunohistochemical analyses have shown increased fibrinogen, glial fibrillary acidic protein (GFAP), and ionized calcium-binding adaptor molecule 1 (Iba1) in the fimbria of NOD mice, indicating decreased BBB integrity as well as increased astrocyte and microglia activation similar to the chronic activation of CNS-resident immune cells found in PMS [168]. For comparison, demyelination, nor astrocyte and microglia activation was not present in the brain of C57Bl/6 J mice. However, elevated fibrinogen, GFAP, and Iba1 was found in the spinal cord of both NOD and C57Bl/6 J mice, suggesting that the pathology in the spinal cord is similar between the two strains [168].

The immune landscape in the NOD-EAE model shares similarities to what has been reported in PMS patients. In this model a 13-fold increase in CD8 + T cells and a 20-fold increase in CD4 + T cells in the spinal cord was reported when comparing the percentage of infiltrates to control mice [169]. This recapitulates the increase in CD8 + T cells observed in PMS patient, without mimicking the marked increase in the CD8 + :CD4 + ratio. Furthermore, B cells and MOG-specific autoantibodies are increased in the NOD-EAE model potentially making this model useful for mimicking and studying the role of mature plasma cells, which are found in the CNS of PMS patients. Additionally, TFH cells have been found to be increased in the spinal cord and immunohistochemical analyses have shown formation of ELFs in the spinal cord, identified as clustering of B cells, CD4 + T cells, and CD35 + FDCs in the chronic stage of disease [165, 169]. This immune landscape, the astrocyte and microglial activation align the NOD-EAE model with the compartmentalized inflammation seen in SPMS.

If EAE is induced in a transgenic immunoglobulin (Ig)H^[MOG]^ NOD mouse strain, the mice develop a severe, progressive disease course accompanied by PMS-like pathology [95]. These mice have lymphocyte infiltration, spinal cord demyelination, as well as structures reminiscent of ELFs observed in SPMS patients, in the brain and cerebellar meninges and brain sulci of immunized IgH^[MOG]^ mice [95, 170]. Additionally, there is an expansion of fibronectin and platelet-derived growth factor receptor (PDGFR) α/β below these ELF-like clusters, indicating the presence of a stromal cell network, characteristic of ELFs [95, 171].

Potential inadequacies of the NOD-EAE model to mimic PMS were recently pointed out by Baker et al. [172]. They highlighted results showcasing the NOD-EAE model as a progressive model stem from asynchronous relapses in individual mice, which they argued could, when plotted as mean disease scores, erroneously imitate a progressive disease evolution [172]. As EAE – like MS – can be quite heterogenous in terms of the onset, disease progression, and severity, it is important to report data regarding disease course on an individual basis (Table 3). Additionally, B cell involvement appears limited or inconsistent compared to human PMS, and the reliance on MOG immunization does not fully reflect the antigenic complexity of MS. These limitations suggest that while the NOD-EAE model is valuable for studying aspects of PMS, its interpretation requires caution.

**Table 3:** The curve conundrum: individual vs. average in EAE disease progression

In studies using experimental autoimmune encephalomyelitis (EAE) to model multiple sclerosis, disease progression is often presented as average clinical scores over time. While this approach offers a simplified overview of group-level trends, it can potentially obscure the substantial variability that exists between individual animals – variability that is especially relevant in models of progressive multiple sclerosis
Individual curves provide a more nuanced view of disease dynamics, revealing differences in onset, peak severity, recovery, and disease duration that are often masked in mean data. They can be particularly useful for identifying subgroups of animals with distinct responses to treatment—information that is critical for understanding therapeutic efficacy
Baker et al. (2019) caution against relying solely on averaged data. From their study of EAE in Non-Obese Diabetic (NOD) mice they found that what appeared to be progressive disease in group averages was, in fact, a relapsing–remitting pattern in many individuals. This misclassification could have been avoided by examining individual disease courses, highlighting the risk of overgeneralization when relying on mean curves alone [172]
However, Buonvicino et al. (2019) immunized a total of 288 NOD mice and found a primary progressive disease course. Only 5.6% of mice had a partial remission phase, and none had an early remission phase. They observed that while all animals eventually reached a severe disease score, the rate of progression varied dramatically, with some mice reaching this endpoint within 12 days and others taking up to 122 days. However, when the individual data is cumulated it presents as a primary progressive pattern [169] That said, averaged data remains valuable. They provide a concise summary of overall trends and are essential for statistical comparisons. In larger cohorts, mean curves with appropriate error bars can also improve clarity and readability, especially when the primary goal is to assess general treatment effects We believe that the most informative and rigorous approach is to present both individual and averaged data. This dual representation ensures that the complexity of EAE progression is preserved while still allowing for statistical interpretation and clear communication of group-level findings

### Biozzi ABH mice – relapsing–remitting to secondary progressive disease course

The Biozzi-EAE model closely mirrors key features of human PMS, particularly SPMS. In this model, spinal cord homogenates are most frequently used as the immunogen. The model exhibits a transition from a relapsing–remitting phase to a chronic progressive stage, accompanied by shifting immune profiles from T to B cell dominance and a dynamic change in BBB integrity [173–175]. The Biozzi-EAE model presents with accumulating demyelination and axonal loss, and in the late chronic phase the demyelinated lesions present with decreased lesional activity (reduction of Iba1 expression) and increased astrogliosis (increase of GFAP expression), imitating features of the inactive lesions found in PMS [173]. Additionally, in the late, chronic stage, mature ELFs form along the meninges in the spinal cord, identified by clusters of B-220 + B cells, Ki67 + cells, CD35 + dendritic cells and presence of CXCL13 [173]. Furthermore, there is evidence suggesting that neuronal loss persists even if relapses and progression are eliminated by tolerization, a process reminiscent of PIRA in human patients (Table 1) [176].

Inducing EAE in old mice from this strain changes the disease course observed in younger mice, secondary progressive EAE (SP-EAE), to primary progressive EAE. This is associated with an increase in T cell presence in WM and GM, increased WM microglia/macrophages, and increased axonal damage. Microglial activation was associated with WM neuronal damage that was more pronounced in aged mice presenting with progressive EAE [177].

Interestingly, administration of a third subcutaneous injection of the immunogen in this model during a period of remission makes it possible to induce a synchronized relapse. Signs of relapse appear approximately one week after injection, allowing for more uniform neurological deficits that correlate with loss of spinal cord nerves [175]. This approach could circumvent some of the concerns raised in Table 3, and furthermore be beneficial when screening novel, neuroprotective drugs which do not act as an immunosuppressant.

However, dissimilarities remain between this model and human pathology. The disease course is accelerated compared to the typically slower heterogeneous progression in humans. The limited involvement of cortical GM and the artificial induction of relapses may also constrain its ability to fully replicate the complexity of human PMS.

### SJL mice – relapsing–remitting and primary progressive disease course

The SJL-EAE model is primarily thought of as a model of RRMS. However, this model can mimic aspects of PMS under specific conditions, such as administration of the Th17 inducing co-adjuvant Curdlan prior to immunization with MOG_92–106_, resulting in demyelinating lesions both in the perivascular space and parenchymal space of the spinal cord, and significant parenchymal T cell and neutrophil infiltration [178, 179].

Ultraviolet B irradiation converts approximately one third of RR-EAE mice to SP-EAE mice. These mice have lesions in both the spinal cord and brain and increases pathological scores in both the brain and spinal cord. Interestingly, mice presenting with SP-EAE have significantly lower numbers of T cells in the meninges, parenchyma, and perivascular space in spinal cord and brain, and increased microglia/monocyte-derived macrophage presence in demyelinating lesions – features that are reminiscent of inactive lesions in PMS pathology [180].

However, dissimilarities include the typically relapsing–remitting baseline course, limited B cell involvement, and rapid disease progression in some induced models (e.g., hyperacute EAE), which diverge from the slower, more compartmentalized progression seen in human PMS. These differences highlight both the model’s utility and its limitations in replicating the full spectrum of PMS pathology.

### A.SW mice – primary progressive disease course

The A.SW-EAE model offers a unique representation of both PPMS and SPMS, with disease courses that include both PMS phenotypes. The model can be induced in both males and females, but females tend to present with increased severity compared to males [152].

A.SW mice immunized with MOG_92–106_ with co-adjuvant present with an EAE that started off as RR and later progress to a progressive disease course with or without relapses, reminiscent of a SPMS-like disease course [87].

A.SW mice immunized with MOG_92–106_ without administration of a co-adjuvant develop ataxic EAE with a progressive disease course with clinical signs becoming evident approximately one-month post-immunization [87, 178]. The majority of these mice present with a progressive disease course from onset and die on average 20 days after disease onset. Some mice have relapses in their progressive disease course, reminiscent of progressive-relapsing MS [87].

Both mice with PPMS-like and SPMS-like disease courses have increased CNS pathology in terms of lesion burden and the presence of plaque-like lesions in both cerebellum and spinal cord, but a markedly decreased fraction of CD3 + infiltrating cells. This reduction in T cells in part recapitulates the decreased inflammation seen in PMS patients [181]. Furthermore, the PPMS-like A.SW mice have heavily increased numbers of neutrophils, macrophages/microglia, and reactive glial cell in the demyelinating lesions, which is another feature shared with PMS. Additionally, it has been found that there is extensive IgG deposition in myelin and endothelial cell specific areas, as well as in plaque margins in both PPMS-like and SPMS-like A.SW mice, features reminiscent of pathological hallmarks of MS brains [181].

If mice are injected with the co-adjuvant PTX, they present with a secondary progressive disease course: the mice initially develop RR-EAE but then transition into a progressive disease course that may include occasional periods of remission and relapse [87].

This model also diverges from human PMS in several key aspects. Unlike the T cell-dominated pathology typical of MS, the A.SW model shows minimal T cell involvement and instead emphasizes antibody- and complement-driven mechanisms. Based on findings from this model of PMS, it has also been suggested that B-1 cells – a T cell independent, natural autoantibody producing, type of B cell – are involved in modulation of progressive EAE in A.SW mice. However, the overall effect of depleting peritoneal B-1 cells on disease course was minor, suggesting that T cell-dependent, IgG-producing B cells have a more impactful role. The dominant role of classic B cells and the limited contribution of B-1 cells also contrast with the broader B cell heterogeneity observed in MS. Additionally, the rapid disease progression and high mortality limit the utility for studying long-term neurodegeneration [182].

## Other animal models of PMS

### The cuprizone model – demyelination with extensive glial activation and neuronal loss

While the EAE model mimics part of the immune mechanisms seen in MS and the subsequent demyelination and lesion formation, this model does not present with the neurodegenerative aspect of the pathology. The cuprizone model is based on oral ingestion of the copper chelator cuprizone, causing metabolic oligodendrocyte injury. It is most often induced in C57Bl/6 mice [183].

The cuprizone model offers a valuable complement to EAE in preclinical PMS research by enabling the study of demyelination and neurodegeneration independent of adaptive immune responses. Demyelination begins in the corpus callosum after three weeks of cuprizone intoxication and becomes significant after five weeks compared to control mice [184, 185]. Microglial activation and axonal loss positively correlate with demyelination and the reproducible lesion localization – primarily in the corpus callosum [186], cerebellar peduncle [186], hippocampus [187], and cortex [184, 185, 188] – mirrors the topographical specificity of MS pathology, including subpial cortical demyelination seen in PMS patients.

In contrast to the EAE model where lesion formation happens stochastically/sporadically in the spinal cord, the lesions are confined to these certain topographical areas in the brain – increasing the reproducibility [189, 190]. The predictability of lesion locations makes the cuprizone model great for investigating de- and remyelinating processes and to evaluate potential remyelinating therapies [151]. Upon cuprizone removal after acute exposure (5 weeks), spontaneous remyelination takes place [187]. However, after chronic cuprizone exposure (12 weeks or more) the regenerative capacity of oligodendrocytes decrease and remyelination is halted, leading to axonal loss, mimicking the neurodegenerative phase of PMS [191].

For a long time, it was believed that the cuprizone model preserves BBB integrity and therefore that T- and B cell infiltration is absent. This was thought to allow investigation of de- and remyelinating processes independent of immune factors [192, 193]. However, more recent studies have shown increased BBB permeability occurring already after three days of cuprizone administration and parallelling the observed demyelination [194]. Furthermore, the density of T cells in corpus callosum is significantly increased after five weeks of cuprizone intoxication which accounts for an increase in both CD4 + as well as CD8 + T cells. Additionally, in the anterior commissure and rostral hippocampus there is a significant negative correlation between myelin density and T cell density, indicating that cuprizone-induced demyelination triggers recruitment and infiltration of T cells to the site of injury. Interestingly, CD8 + T cells have been found to outnumber CD4 + T cells in the cuprizone model, which is in line with the CD8 + dominated lesions seen in PMS patients [184].

### Combined cuprizone/EAE model – widespread demyelination and infiltration of CNS

Intoxicating animals with cuprizone prior to EAE induction can affect the immunoreactivity and disease course. This combined cuprizone/EAE model bridges neurodegeneration and immune-mediated inflammation, capturing key aspects of PMS. If cuprizone and EAE are applied sequentially, such that the animals receive a non-intoxicated diet for two weeks before EAE immunization, lesions develop in the forebrain as well as the cervical, thoracic, and lumbar parts of the spinal cord [195, 196]. Furthermore, this model allows for enhanced microglial and macrophage activation [195], and increased immune cell infiltration including T cells, microglia [197], granulocytes [198] and neutrophils [195].

The combination of cuprizone and EAE promotes T cell infiltration to the topographical lesion sites, which is not seen in the cuprizone nor the EAE models alone, where T cell infiltration is more diffuse [199]. This suggests that neurodegenerative processes may act as initiating factors in lesion formation and contribute to the recruitment of peripheral immune cells [196, 200, 201]. The lesions in this model are characterized by oligodendrocyte death and axonal injury in close proximity to perivascular infiltrates. This is reminiscent of histopathological features of type III lesions – a lesion type associated with the progressive stages of MS [198, 201, 202].

However, outcomes vary depending on timing and duration of cuprizone exposure. Some studies have reported an immunosuppressive effect of cuprizone when given prior to immunization with MOG_35–55_. However, if cuprizone intoxication is ongoing at the time of immunization, EAE is inducible, although with a milder disease course [198, 203].

### Lysolecithin-induced demyelination – focal detergent-driven demyelination

The lysolecithin model provides a highly controlled, non-immune-driven system for studying demyelination and remyelination, making it especially valuable for investigating regenerative therapies for PMS [151]. This model is based on focal injections of lysolecithin, often into the dorsal or ventrolateral funiculi of the spinal cord inducing focal lesions in the spinal cord [204]. Its focal lesion induction and predictable remyelination timeline allow for precise assessment of myelin repair mechanisms [205]. While it lacks the inflammatory component of MS, this model is ideal for isolating and testing remyelination-promoting strategies in a reproducible and anatomically targeted manner.

Interestingly, if lysolecithin-treated mice are injected with CSF from untreated PPMS patients, the remyelination process is inhibited, and there is an increase in microglial activation and astrogliosis, indicating a presence of factors in the CSF of PPMS patients causing a delay in remyelination [206].

Dehghan et al. established an optimized lysolecithin model, that could potentially be useful for investigating neurodegenerative mechanisms and related treatment efficacy. In this model, agarose-gel loaded lysolecithin is applied to the optic nerve, causing long-term demyelination and axonal loss followed by degeneration of retinal ganglion cells [207].

Overall, the lysolecithin model is highly advantageous for PMS research due to its controlled induction of focal demyelination, predictable repair kinetics, and capacity to dissect remyelination mechanisms in isolation from adaptive immune responses. However, its major limitation lies in the absence of chronic inflammation, neurodegeneration, and immune-mediated pathology. So, while it provides valuable insight into the cellular and molecular mechanisms of remyelination failure, it does not fully recapitulate the inflammatory and neurodegenerative features of PMS.

### TMEV – virally-induced chronic, progressive demyelinating disease

Due to the strong epidemiological and mechanistic link between MS and Epstein–Barr virus (EBV), viral models have gained renewed interest as tools to investigate how persistent infection may trigger or sustain chronic neuroinflammation. One of the most extensively characterized models is Theiler’s murine encephalomyelitis virus–induced demyelinating disease (TMEV-IDD), where intracerebral infection of SJL mice with TMEV causes a late-onset demyelinating disease [27]. A subgroup of low neurovirulence TMEV known as Theiler’s Original (TO) induces a biphasic disease – first acute poliomyelitis followed by chronic, immune-mediated demyelination, neurodegeneration, and spinal cord atrophy. This chronic stage shares several pathological features with progressive MS (PMS), including ongoing microglial activation, diffuse axonal injury, and compartmentalized inflammation within the CNS [208, 209]. Importantly, the TMEV-TO model exhibits epitope spreading from viral to myelin antigens and progressive axonal loss, thereby providing experimental support for the hypothesis that viral infection may initiate autoimmunity through bystander activation and molecular mimicry [210]. This phenomenon is particularly relevant considering recent data demonstrating that EBV infection is a necessary risk factor for MS and that cross-reactive immune responses between EBV antigens and CNS proteins may contribute to disease initiation [211, 212]. While TMEV is not homologous to EBV, both models share key pathogenic principles: viral persistence, chronic intrathecal immune activation, and secondary autoimmune responses.

The TMEV model, its immune landscape, and similarities to and differences from MS is extensively reviewed by Pike et al. [209]. B cells play a central role in TMEV-IDD, particularly during the chronic phase. Persistent infection drives intrathecal B cell accumulation, local antibody production, and formation of lymphoid-like aggregates within the CNS. However, the aggregates are lacking the germinal centre-like structure found in ELFs. [213].

CD8⁺ T cells are essential for viral control during the acute phase, yet incomplete clearance permits viral persistence in macrophages/microglia and astrocytes, creating a state of smouldering-like neuroinflammation [214, 215]. Over time, the inflammatory milieu shifts from predominantly antiviral to autoimmune, with CD4⁺ Th1/Th17 responses targeting myelin antigens [216, 217]. Chronic microglial activation, mitochondrial injury, and progressive axonal transection dominate the late stage, closely resembling mechanisms implicated in PMS rather than relapsing MS [218].

Thus, TMEV-IDD provides a unique platform to study how viral persistence may drive chronic, compartmentalized CNS inflammation and progressive neurodegeneration. In contrast to classical EAE, which is typically driven by peripheral T cell priming against defined myelin peptides, TMEV incorporates: a viral trigger, epitope spreading, intrathecal B cell responses, and progressive axonal loss independent of relapses. These features make the model particularly relevant for investigating EBV-associated mechanisms and the transition from virus-driven immune activation to progressive MS-like pathology [219].

**Fig. 2:**
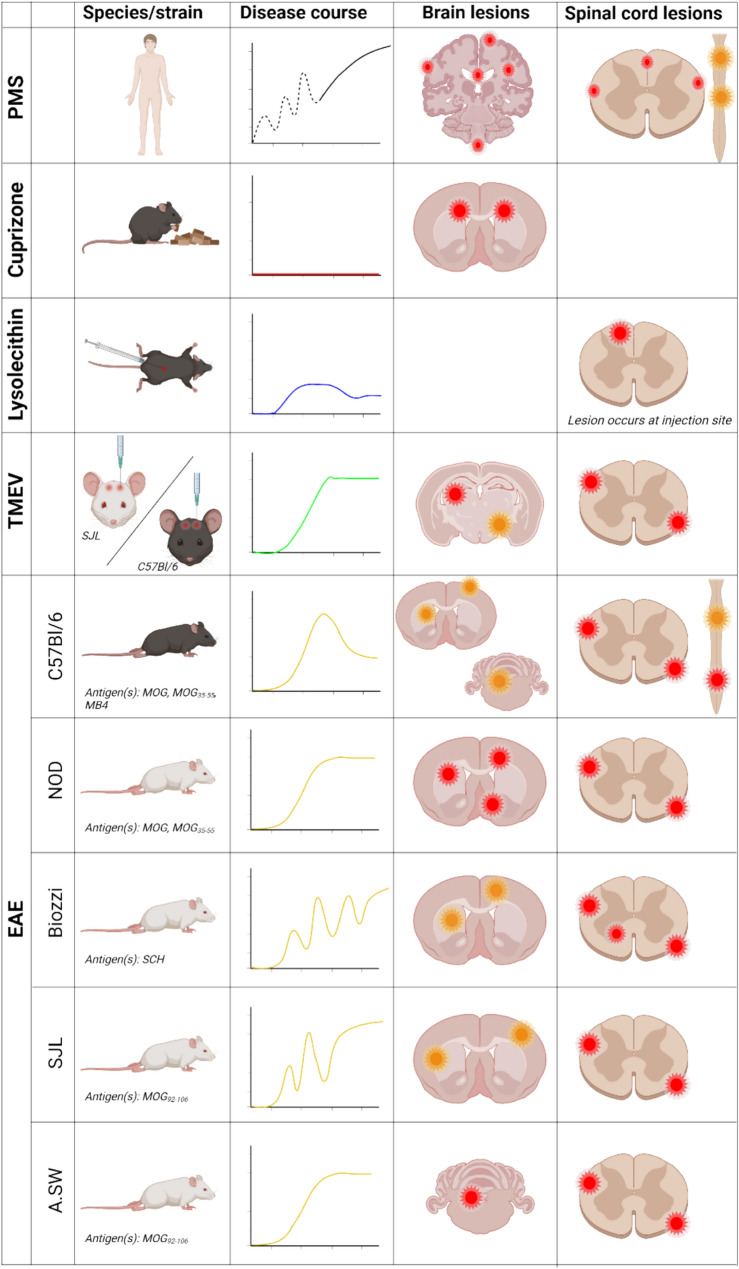
Illustrative disease progressions patterns and lesion localization in brain and spinal cord in PMS and animal models of PMS. PMS, progressive multiple sclerosis; EAE, Experimental autoimmune encephalomyelitis; TMEV, Theiler’s Murine Encephalomyelitis VirusIllustration of commonly used animal models of demyelination and neuroinflammation compared with human PMS. Each model differs in disease course, immunopathology, and lesion localization within the brain and spinal cord. Red areas indicate predominant lesion sites, orange areas indicate less frequent lesions sites. Time-course plots represent the relative onset and duration of clinical disease worsening

Figure 2 provides an overview of the disease progressions patterns and common lesion sites of these most commonly used animal models compared to PMS.

## Discussion

PMS remains one of the most challenging neurological diseases to both study and treat. While DMTs have transformed the outlook for RMS, to this day, therapeutic success in PMS is far more limited. This gap reflects, in part, the scarcity of animal models that recapitulate the complex, compartmentalized inflammation and neurodegeneration seen in human PMS. The animal models reviewed here – particularly murine EAE – have been instrumental in understanding MS immunopathology, validating therapeutic targets, and enabling drug development and approval. Yet, their ability to reproduce the slow, progressive accumulation of disability characteristic of PMS is limited, highly variable, and model dependent.

### Strengths of current models

Across multiple EAE variants, key aspects of PMS biology can be recapitulated. Certain strains and induction protocols (e.g., C57Bl/6 with MBP–PLP fusion protein, NOD mice, Biozzi ABH mice) capture hallmark features such as ELFs, chronic microglial activation, iron rim–like lesions, and a shift from peripheral infiltration to compartmentalized CNS inflammation. Models such as Biozzi ABH and aged C57Bl/6 can also mimic the clinical transition from relapsing–remitting to secondary progressive disease.

Despite criticism, the EAE model remains a cornerstone of preclinical MS research. Most approved RMS therapies have shown efficacy in EAE, and although translation to PMS-specific treatments is less direct, these models still provide critical mechanistic insights. The diversity of established models offers flexibility to investigate specific disease mechanisms, from neurodegeneration to remyelination.

### Limitations and translational gaps

Despite these strengths, there are significant limitations to the current models that constrain their predictive and translational value. Limitations specific to the individual models highlighted in this review are mentioned at the end of the respective sections.

In general, active EAE relies on immunization with an antigen and adjuvants, which departs from the spontaneous, multifactorial etiology of human MS. Furthermore, lesion patterns in EAE are predominantly spinal cord–centric, with limited cortical GM involvement. The timeline for the accumulation of physical symptoms that follows the lesion formation is extremely exaggerated, with animals experiencing paralysis or death anywhere from 15 days post-immunization to a couple of months after immunization. As highlighted in Table 3, averaged disease curves can obscure individual disease trajectories, leading to potential misclassification of relapsing patterns as progressive courses.

Additionally, the immune pathology of these models is often limited. Murine models rarely replicate the elevated CD8 + :CD4 + T cells ratio, that is highly characteristic of PMS lesions. The contribution of B cells is often less pronounced, or mechanistically distinct, compared with that observed in human disease.

Moreover, most of the animal models replicate autoimmune inflammation, but only few aim at modelling CNS-compartmentalized inflammation that is a hallmark of PMS. The cuprizone model is a more frequently used model, that allows for the investigation of de- and remyelination and neurodegeneration independent of adaptive immunity. A less explored model system, that has potential of modelling the inside-out paradigm of MS or the CNS-compartmentalized inflammation linked to PMS, is Diphtheria Toxin A induced oligodendrocyte ablation models [220, 221]. These models allow for oligodendrocyte-selective cell death, hereby making it possible to study de- and remyelination. This model was shown to have widespread CNS WM demyelination as well as motor symptoms such as ataxia and tremor, starting around 5 weeks after induction [221]. Demyelination has been shown to be accompanied by increased microglia/macrophage activation, decreased myelin clearance, and limited remyelination [222]. These mice have been shown to recover from their clinical symptoms, but interestingly in the long term they develop a secondary fatal immune-mediated disease, characterized by demyelination and T cell infiltration [223].

However, animal models of human diseases rarely recapitulate the entire disease including etiology and pathology. This does not make them invaluable, as animal models can be a great tool to investigate specific aspects of a disease. Therefore, it is important to be mindful about choosing the appropriate model depending on which aspects of PMS pathology is to be investigated.

### Opportunities for refinement

With that said, we should not stop searching for and continuously aim at developing refined animal models that will mimic the key aspects of PMS. The fact that only one drug has been approved for treatment of PMS highlights the lack of preclinical tools that can both increase our understanding of the complex pathology and aid in approvals of future therapies. Model refinement should aim to integrate immune-driven progression with neurodegeneration and remyelination failure in a reproducible, measurable manner.

Combining models, such as EAE and cuprizone, has great potential for coupling neurodegeneration with inflammation and lesion formation. Additionally, when exploring new animal models of PMS, it is important to remain aware of the impact of age, sex, and strain, as these parameters can steer the models towards PMS-like pathology. This emphasizes the importance of reporting detailed protocols when publishing experimental data. Substrains of animals are often not specified, although known to affect model robustness [224, 225]. Clear and concise reporting of protocols would help unify and streamline the currently highly variable EAE field, easing comparison and reproducibility across studies and laboratories. Moreover, optimizing and clearly describing induction protocols may facilitate the establishment of models with chronic, progressive disease courses.

This review has mainly focused on murine EAE models of PMS, as this is one of the most widely used models in preclinical MS research. However, EAE is inducible in multiple species. In Dark Agouti rats immunization with MOG_1–125_ produces a chronic relapsing disease course with chronic demyelination [226]. Lewis rats are known to present with RR-EAE, but backcrossing them to a MHC-congenic strain leads to acute progressive EAE or SP-EAE [227, 228]. Similarly to the murine models, lesions in these rat models are primarily located in the spinal cord, and they share many of the same limitations. Combined with the increased costs of housing rats and the scarcity of genetically modified substrains, this helps explain why murine models have historically and remain most used. Nevertheless, a focal, targeted EAE model can be induced in Lewis rats: sensitizing rats with a sub-threshold amount of MOG peptide before intracerebrally administering cytokines triggers lesions that appear at the site of injection [229]. This approach enables lesion formation in the brain and, unlike the lysolecithin model, incorporates an immune component. Another model based on the adoptive transfer of β-synuclein-specific T cells into Lewis rats leads to clinical symptoms including paresis, ataxia and head-tilting [230]. These β-synuclein-specific T cells mainly infiltrate the cortical GM, reflecting an important hallmark of MS. Moreover, β-synuclein-specific T cells increase in the blood of patients with PMS, further increasing the translatability potential of this model [230].

Despite these refinements, rodent models still only partially capture the complexity of PMS pathology, prompting interest in models that more closely resemble human disease.

NHP models, particularly the common marmoset, offer significant advantages in preclinical PMS research due to their close immunological and neuroanatomical resemblance to humans [231]. These models exhibit key pathological features of PMS, including cortical demyelination, meningeal inflammation, and CD8 + T cell-mediated neurodegeneration, which are rarely captured in rodent models [232, 233]. Moreover, NHPs allow for the evaluation of human-specific therapeutics and immune responses. However, their use is constrained by ethical considerations, high costs, limited availability, and variability in disease induction, which collectively hamper scalability and reproducibility in large-scale studies [234].

However, as evident from this review, the vast majority of animal model systems of MS are often T cell centric. This leaves behind an unmet need for preclinical model systems that allow for the scrutinization of B cells in MS pathology as well as pharmacodynamics especially in the wake of the successful use of anti-CD20 antibodies in MS disease management, and the promising Phase III trials of the BTK inhibitor Fenebrutinib as the potentially first treatment approved for both RMS and PPMS [235].

## Future perspectives

Next-generation PMS models should strive for greater standardization of protocols, and integration of immune, glial, and neurodegenerative components, reflecting the dynamic interplay seen in patients. Applying clinical frameworks such as PIRA and relapse-associated worsening (RAW) to preclinical disease monitoring may improve translational relevance. Incorporating longitudinal imaging, standardized lesion quantification, and dual reporting of group and individual disease courses will further strengthen the field.

## Concluding remarks

No single animal model fully recapitulates PMS. However, when protocols are synergized, and used with awareness of their respective strengths and limitations, existing models – especially in combination – can become powerful tools for understanding disease mechanisms and identifying candidate therapeutics [232]. While this review focuses on active EAE, complementary approaches including transgenic, knockout, toxin-induced, and viral models all have unique contributions to make. Strategic use of this expanded toolbox, coupled with rigorous reporting and refined experimental design, will be essential to bridge the translational gap between bench and bedside in PMS research.

## Data Availability

No datasets were generated or analysed during the current study.

## Abbreviations

BBB: Blood-brain barrier
CH-EAE: Chronic experimental autoimmune encephalomyelitis
CIS: Clinically isolated syndrome
CNS: Central nervous system
CSF: Cerebrospinal fluid
DMT: Disease-modifying therapy
EAE: Experimental autoimmune encephalomyelitis
ELFs: Ectopic lymphoid follicles
FDC: Follicular dendritic cell
GFAP: Glial fibrillary acidic protein
GM: Grey matter
Iba1: Ionized calcium-binding adapter molecule 1
IgG: Immunoglobulin G
MBP: Myelin basic protein
MOG: Myelin oligodendrocyte glycoprotein
MRI: Magnetic resonance imaging
MS: Multiple sclerosis
NHP: Non-human primate
NOD: Non-obese diabetic
PIRA: Progression independent of relapse activity
PDGFR: Platelet-derived growth factor receptor
PLP: Proteolipid protein
PMS: Progressive multiple sclerosis
PPMS: Primary progressive multiple sclerosis
PTX: Pertussis toxin
RAW: Relapse-associated worsening
RMS: Relapsing multiple sclerosis
RR-EAE: Relapsing–remitting experimental autoimmune encephalomyelitis
RRMS: Relapsing–remitting multiple sclerosis
SP-EAE: Secondary progressive experimental autoimmune encephalomyelitis
SPMS: Secondary progressive multiple sclerosis
TFH: T follicular helper
Th: T helper
TMEV: Theiler’s murine encephalomyelitis virus
TMEV-IDD: Theiler’s murine encephalomyelitis virus induced demyelinating disease
TO: Theiler’s Original
WM: White matter
